# A machine learning model for the early diagnosis of bloodstream infection in patients admitted to the pediatric intensive care unit

**DOI:** 10.1371/journal.pone.0299884

**Published:** 2024-05-01

**Authors:** Felipe Liporaci, Danilo Carlotti, Ana Carlotti

**Affiliations:** 1 Department of Pediatrics, Division of Pediatric Critical Care Medicine, Ribeirão Preto Medical School, University of São Paulo, São Paulo, Brazil; 2 Institute of Mathematics and Statistics, University of São Paulo, São Paulo, Brazil; Shiraz University of Medical Sciences, ISLAMIC REPUBLIC OF IRAN

## Abstract

Bloodstream infection (BSI) is associated with increased morbidity and mortality in the pediatric intensive care unit (PICU) and high healthcare costs. Early detection and appropriate treatment of BSI may improve patient’s outcome. Data on machine-learning models to predict BSI in pediatric patients are limited and neither study included time series data. We aimed to develop a machine learning model to predict an early diagnosis of BSI in patients admitted to the PICU. This was a retrospective cohort study of patients who had at least one positive blood culture result during stay at a PICU of a tertiary-care university hospital, from January 1^st^ to December 31^st^ 2019. Patients with positive blood culture results with growth of contaminants and those with incomplete data were excluded. Models were developed using demographic, clinical and laboratory data collected from the electronic medical record. Laboratory data (complete blood cell counts with differential and C-reactive protein) and vital signs (heart rate, respiratory rate, blood pressure, temperature, oxygen saturation) were obtained 72 hours before and on the day of blood culture collection. A total of 8816 data from 76 patients were processed by the models. The machine committee was the best-performing model, showing accuracy of 99.33%, precision of 98.89%, sensitivity of 100% and specificity of 98.46%. Hence, we developed a model using demographic, clinical and laboratory data collected on a routine basis that was able to detect BSI with excellent accuracy and precision, and high sensitivity and specificity. The inclusion of vital signs and laboratory data variation over time allowed the model to identify temporal changes that could be suggestive of the diagnosis of BSI. Our model might help the medical team in clinical-decision making by creating an alert in the electronic medical record, which may allow early antimicrobial initiation and better outcomes.

## Introduction

Bloodstream infection (BSI) is defined by the presence of positive blood cultures in patients with systemic signs of infection [1]. BSIs are associated with increased morbidity and mortality in pediatric critically ill patients and higher hospital costs [2]. They can be community-acquired or acquired in a hospital or other healthcare facility [1, 3]. In the pediatric intensive care unit (PICU) setting, there is evidence that the presence of invasive devices, including central lines, urinary catheters and endotracheal tubes significantly increases the risk of health-care associated BSIs [4].

The incidence of health-care associated BSIs varies around the world. The reported rate of central line-associated BSIs is 1.4 per 1000 catheter-days in PICUs from the USA [5]. However, a systematic review and meta-analysis that included data from 79 PICUs all over the world reported a median incidence of 5·9 per 1000 catheter-days (range 2·6–31·1) [2]. In addition, mortality rate was more than twice as high for patients who developed a central line-associated BSI in the PICU compared with those who were BSI-free (15% vs. 7%) [6, 7].

Early detection and adequate antimicrobial therapy are essential to improve the outcome of BSIs [8]. Nevertheless, blood culture results may be delayed for several hours or days and the clinicians’ ability to predict BSIs may be limited, which may consequently delay their diagnosis and appropriate treatment [9]. In recent years, numerous studies have applied machine learning models to predict BSIs in critically ill patients [9–12], but data in pediatric patients are limited [12, 13] and neither study included time series data.

Machine learning is a subset of artificial intelligence that refers to the development and application of computer algorithms to complete a task by learning from patterns in the data, using annotated data or not. Machine learning techniques can be supervised, unsupervised and semi-supervised. In supervised learning, labeled datasets are used to train the model to correctly classify the data and the trained model is subsequently validated on additional datasets to evaluate the performance of the algorithm. Unsupervised machine learning works with unlabeled data and is used to find patterns in the data and group them according to the similarities in the data points. Semi-supervised learning uses a small labeled dataset to classify a larger unlabeled subset of the data. Supervised models are commonly used to predict an outcome while unsupervised learning is frequently used for clustering and phenotyping [14, 15].

We aimed to develop a supervised machine learning model using demographic, clinical and laboratory data collected on a routine basis to predict an early diagnosis of BSI in patients admitted to the PICU.

## Methods

This was a retrospective cohort study conducted at a 16-bed medical-surgical PICU of Hospital das Clínicas of Ribeirão Preto Medical School, University of São Paulo. The study was approved by the Institutional Research Ethics Board on December 17, 2021 (#5174982/2021). The informed consent form was waived because of the retrospective nature of the study. Patients’ data were accessed on April 14, 2022 for research purposes. All individual participants were anonymized by being labeled by numbers on the data collection sheet.

All patients aged 0 to 18 years admitted to the PICU from January 1^st^ to December 31^st^ 2019, who had at least one positive blood culture result during PICU stay were eligible for the study. Patients with positive blood culture results with growth of contaminants and patients with incomplete data were excluded.

### Data collection

Data were collected from patients’ electronic medical records, including:

- Demographic data: age, gender, weight, weight-for-age z score, height, body mass index (BMI) and BMI z score for age.
- Presence of comorbidities (yes or no): undernutrition/ obesity, congenital heart disease, neoplasia, neurological, pulmonary, gastrointestinal, liver, rheumatological or urinary tract disease.
- Disease severity assessed by PRISM III score in the first 4 hours after admission to the PICU [16].
- Organ dysfunction assessed by PELOD score in the first 48 hours after admission to the PICU [17].
- Use of intravenous devices (central venous catheter, peripheral venous catheter, peripherally inserted central catheter, totally implantable central venous catheter), duration of use until blood culture collection and puncture site (femoral or not).
- Treatment data: use of total parenteral nutrition and duration of use until blood culture collection, postoperative period (yes or no), bone marrow or solid organ transplantation (yes or no), use of chemotherapy (yes or no).
- Laboratory data and vital signs collected for the study were obtained 72 hours before and on the day of blood culture collection, and they included:
- Laboratory data: C-reactive protein concentrations, complete and differential white blood cell counts and platelet counts.
- Vital signs: heart rate, respiratory rate, blood pressure, temperature, oxygen saturation. Philips IntelliVue MX450 was used for vital signs monitoring.

Table 1 shows the normal reference values of laboratory data.

**Table 1:** 

**Variable**	**Normal values**
C-reactive protein (mg/dL)	0–0.5
White blood cell count/mm^3^	6600–15600
Myelocytes/mm^3^	0
Metamyelocytes/mm^3^	0
Bands/mm^3^	0–400
Neutrophils/mm^3^	1500–7400
Lymphocytes/mm^3^	3200–11200
Platelet count/mm^3^	240000–550000

### Data processing

The data collected from all patients were initially pre-processed for an exploratory analysis to verify if there were patterns recognizable by the medical team in the values found before and after the onset of infection, detected by the reference values.

**Fig 1:**
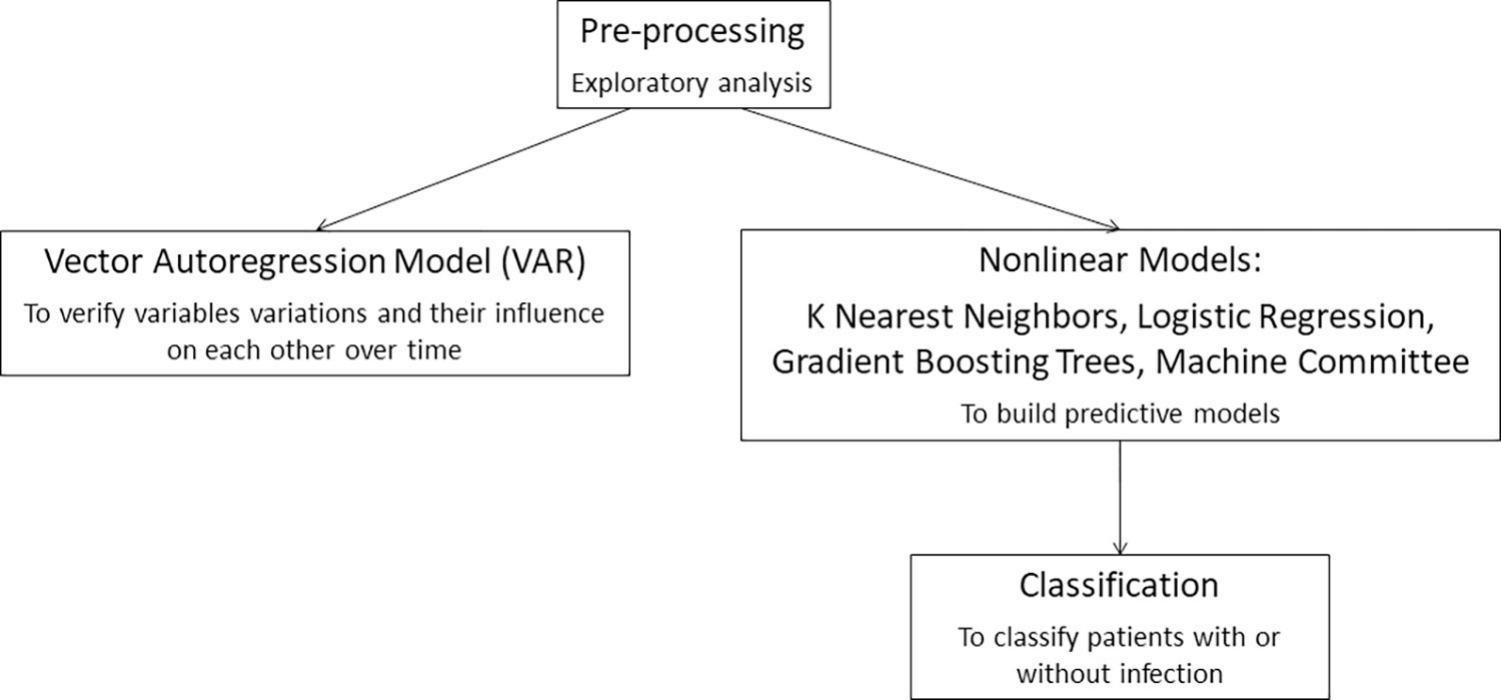
Data processing.

Two alternatives were tested for the development of models that could contribute to physicians’ decision-making: the first one considered the variations of laboratory test results and vital signs measurements as time series. This was done by using algorithms such as vector autoregression model (VAR) [18] to model how the variables behave and how they influenced each other over time. The second approach was to build predictive models using nonlinear models [19, 20] such as K nearest neighbors, logistic regression, gradient boosting trees and machine committees, which teach several different models to quantify the problem and subsequently create a “committee” that votes for the outcome. Then, there is another model that learns the estimates of these various models to make its decision. The strategy employed aimed to use these models to classify patients with or without infection (Fig 1).

### Model development

Demographic and clinical data, such as age, weight, height, body mass index, presence of comorbidities and use of parenteral nutrition are constant variables and, therefore, they were included only once for each patient. Vital signs and laboratory data were divided into two groups for model training and validation according to the measurement time: 72 hours before blood culture collection (at this time the patient did not have BSI) and on the day of blood culture collection (which subsequently yielded growth of a micro-organism and, therefore, BSI was diagnosed), so each patient was her/his own control. The dependent variable was BSI and all the others were the independent variables. The lines of the matrix of the independent variables were the patients, and the columns included the constant variables and those whose values changed over time, from 72 h before to the day of blood culture collection. The data were classified as 0, when the measurements were performed 72 h before, or as 1, if the measurements were performed on blood culture collection day. Thus, it was expected that the model would be able to separate patients into infected or not, based on the variations of these measurements over time. The patterns and variations of these variables between the two moments in time allow us to test the hypothesis that specific combinations are more likely to happen in non-infected patients and other combinations are more likely to appear in patients with BSI.

The variables included in the models are shown in Table 2.

**Table 2:** Variables included in the models.

Variable	Description	Transformation
Age	Boolean variable	Classified as 0 if more than one year or 1 if less or equal one year.
Weight (kg)	Continuous variable	N/A
Height (m)	Continuous variable	N/A
Weight z-score	Continuous variable	N/A
Body mass index z-score	Continuous variable	N/A
Comorbidity	Boolean variable	Classified as 0 if present or 1 if absent.
Intravenous access device	Categorical variable related to the device type (double lumen central venous catheter, peripherally inserted central catheter, peripheral venous catheter, totally implantable central catheter).	Each possible type of device became a dummy variable for the model. Thus, there are as many columns in the final data as there are variations in the original column. Each dummy column receives 0 or 1. If this device was not used on the patient, the data was classified in the device dummy column as 0 or 1 if the opposite was true.
ΔT IV-line insertion to blood culture collection (days)	Continuous variable	N/A
Lymphopenia	Boolean variable	Classified as 0 if more than or equal to 1000/mm^3^ or 1 if less than 1000/mm^3^.
Duration of total parenteral nutrition until blood culture collection date (days)	Continuous variable	N/A
C-reactive protein (mg/dL)	Continuous variable	The values of this variable for each patient were obtained 72 hours before and on the day of blood culture collection. An additional column was generated with a value of 1 if the measurement was higher than the reference values or 0 if within the reference values.
White blood cell count (/mm^3^)	Continuous variable	The values of this variable for each patient were obtained 72 hours before and on the day of blood culture collection. An additional column was generated with a value of 1 if the measurement was outside the reference values or 0 if within the reference values.
Myelocytes (/mm^3^)	Continuous variable	The values of this variable for each patient were obtained 72 hours before and on the day of blood culture collection. An additional column was generated with a value of 1 if the measurement was outside the reference values or 0 if within the reference values.
Metamyelocytes (/mm^3^)	Continuous variable	The values of this variable for each patient were obtained 72 hours before and on the day of blood culture collection. An additional column was generated with a value of 1 if the measurement was outside the reference values or 0 if within the reference values.
Bands (/mm^3^)	Continuous variable	The values of this variable for each patient were obtained 72 hours before and on the day of blood culture collection. An additional column was generated with a value of 1 if the measurement was outside the reference values or 0 if within the reference values.
Neutrophils (/mm^3^)	Continuous variable	The values of this variable for each patient were obtained 72 hours before and on the day of blood culture collection. An additional column was generated with a value of 1 if the measurement was outside the reference values or 0 if within the reference values.
Lymphocytes (/mm^3^)	Continuous variable	The values of this variable for each patient were obtained 72 hours before and on the day of blood culture collection. An additional column was generated with a value of 1 if the measurement was outside the reference values or 0 if within the reference values.
Platelets (/mm^3^)	Continuous variable	The values of this variable for each patient were obtained 72 hours before and on the day of blood culture collection. An additional column was generated with a value of 1 if the measurement was outside the reference values or 0 if within the reference values.
Vital signs (heart rate, respiratory rate, blood pressure, O_2_ saturation, temperature)	Continuous variable	The values of these variables for each patient were obtained 72 hours before and on the day of blood culture collection. There are several daily readings for all variables. A list was created for all daily readings and four values were generated for all daily readings for each patient: the mean value, the standard deviation, the minimum and the maximum values for the readings of the particular day.

### Model training and testing

Model training followed the best practice of separating data into training and testing sets. The training data are used by the models to learn patterns from the data and to create hyperplanes or decision trees capable of segmenting positive and negative results. Validation or testing data are those that are unknown to the models in the training phase and are used to assess the performance of the model, imitating a situation as close as possible to reality. The segmentation of the testing and training data is repeated several times, using the cross-fold validation method, in which the individual data are randomly assigned to these sets repeatedly. The ratio of the testing and training dataset, for each iteration of the cross-fold validation was always 70%/30%. This means that for each random split of the dataset, 70% of the data was assigned to the training dataset and 30% to the testing dataset. Therefore, the dataset was not imbalanced since the dataset was split into training and testing data subsets in a random fashion, always maintaining the proportion of 50% of zeroes (false) and 50% of ones (true).

Different models trained with different machine learning algorithms were tested. The implementation of all used models is available in the scikit-learn library in Python [21]. The first model is known as a machine committee. A machine committee is a series of models that have two distinct and complementary functions. The first group is comprised of models that are trained in the dataset and make guesses about the data in the same training dataset. There is a model that works as a meta classifier. This model learns the weights it should assign to the prediction of each model, given their accuracy in the training dataset. This whole ensemble is called the committee. In the testing dataset, the models previously trained that belong to the first group assign probabilities for each case. Then, given the probabilities of the models, the meta classifier chooses which models should be “trusted” and it makes therefore its guess about the class of each data point in the testing dataset [22].

The second model is a pipeline in which, at first, a logistic regression with Lasso selects the main variables that should be chosen by the model and, subsequently, a model that employs the gradient boosting decision tree algorithm uses only these variables for its classification. The first step, which is feature selection, can help improve the model’s performance in some cases, even if they are not mandatory to be done. Since the dataset is small, it was considered a valid approach to be tested alongside other approaches. The models are run in a linear sequence. For each iteration, a logistic regression is run and finds the best variables for that specific iteration. Then, it follows and uses this information to inform the following model the variables that should be considered. Each iteration might produce different variables, depending on the random split between training and testing dataset.

The third model is based on the logistic regression algorithm. The algorithm is a statistical method used to classify datasets in one of two classes, such as healthy or sick, true or false. A sigmoid function is used, so for each data point, after training, a function assigns a value between 0 and 1, or 0 and 100%, to the specific instance of the data being analyzed. The formula is shown below [23]:

$$f(x)=L/1+e^{-k(x‐x_{0})}$$

Where: *f* (x) is the output of the function L is the curve’s maximum value k is the logistic growth rate or steepness of the curve x is a real number x_0_ is the x value of the sigmoid midpoint

The fourth model is based on the gradient boosting decision tree algorithm. A decision tree model uses multiple decisions thresholds to assign a data point to one of possible classes. After each iteration, the training dataset is evaluated considering some of the variables available to create the decision tree [24]. The variables have weights and thresholds that allow the model to assign a probability that a data point belongs to a specific class according to its values. In this study, all of the variables were considered by the model more or less useful in determining if a reading from a patient indicates or not that the patient has BSI.

**Fig 2:**
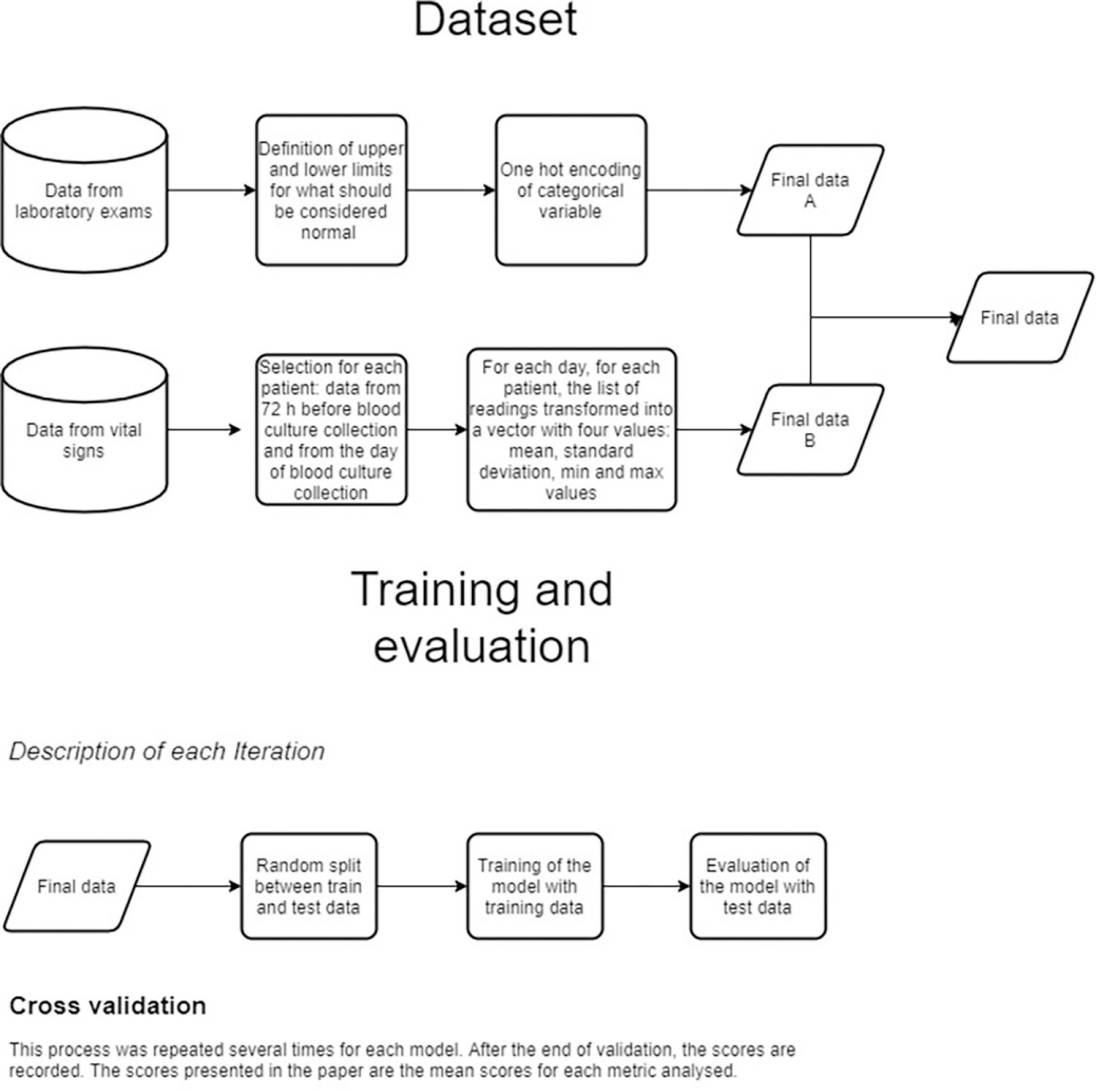


Fig 2 shows the flow diagram of the study.

### Statistical analysis

Descriptive analysis was performed using GraphPad Prism version 8 (GraphPad Software, San Diego, CA, USA). Continuous variables were expressed as median (range). Categorical variables were expressed as number (%). As our dataset was relatively small, the data in our study did not undergo normalization, which could lead to overfitting and possibly data leakage between training and testing datasets. We aimed to verify the ability of the model to identify the thresholds considering the absolute values; thus, data normalization could lead to artificial results and compromise the actual ability of the model to classify the patients. Models’ classification performances were assessed by accuracy, precision, sensitivity and specificity. The modeling and statistical analyses were made using Sklearn package version 0.19, Python programming software version 3.6 (Python software foundation).

## Results

Over the study period, 500 patients were admitted to the PICU; 104 patients had BSI and 76 were included in the study. Patients with a positive blood culture result with growth of contaminants (n = 15) and those with missing data (n = 13) were excluded. A total of 8816 data were processed by the models, including: demographic data (n = 532), laboratory data (n = 1672), clinical data (n = 6080), severity and organ dysfunction scores (n = 152), intravenous device use (n = 76) and treatment data (n = 304). Table 3 shows the demographic, clinical and laboratory data of the study population. Approximately two thirds of patients (49/76) aged less than one year. Most patients had comorbidities; congenital heart disease was the commonest underlying illness and almost one-fifth of the patients were undernourished. The majority of isolates belonged to the genus *Staphylococcus*. Eleven patients (14.5%) were receiving total parenteral nutrition for a median time of 13 days (range 1–790 days). Nine patients (11.8%) were receiving chemotherapy. Double lumen central venous catheters were the most frequent devices, followed by peripherally inserted central catheters; 11 of 35 double lumen central venous catheters and 6 of 18 peripherally inserted central catheters were placed via the femoral vein. Twenty patients (26.3%) were in the post-operative period following cardiac surgery (n = 10), neurosurgery (n = 5) or pediatric surgery (n = 5).

**Table 3:** Demographic, clinical and laboratory data of the study population.

Variables	Study population (n = 76)
Age (months)	4 (0–192)
Weight (kg)	5.1 (2.2–94)
Male gender	37 (52.8)
Weight z score for age	0 (-3–2)
Height (m)	0.55 (0.37–1.67)
Body mass index (kg/m^2^)	15.4 (7.1–34.5)
Body mass index z score for age	0 (-3–3)
Comorbidities Undernutrition Obesity Congenital heart disease Neurological disease Chronic lung disease Gastrointestinal disease Hematologic neoplasia Solid tumor Liver disease Rheumatological disease Urinary tract disease None	14 (18.4) 10 (13.1) 28 (36.8) 9 (11.8) 7 (9.2) 7 (9.2) 6 (7.9) 4 (5.3) 3 (3.9) 2 (2.6) 1 (1.3) 8 (10.5)
Pathogen isolates *Staphylococcus epidermidis* *Staphylococcus hominis* *Staphylococcus aureus* *Klebsiella pneumoniae* *Streptococcus spp*. Other	17 (22.4) 10 (13.2) 9 (11.8) 7 (9.2) 3 (3.9) 30 (39.5)
PRISM III	10.5 (0–33)
PELOD	1 (0–22)
Intravenous access device Double lumen central venous catheter Peripherally inserted central catheter Peripheral venous catheter Totally implantable central venous access Umbilical catheter	35 (46.1) 18 (23.6) 18 (23.6) 4 (5.3) 1 (1.3)
Femoral site	17 (22.3)
ΔT IV-line insertion to blood culture collection (days)	4.5 (0–180)
C-reactive protein (mg/dl) T0 T1	3.1 (0–42.8) 9 (0–45.2)
White blood cell count /mm^3^ T0 T1	10650 (0–37400) 11250 (100–44300)
Myelocytes/ mm^3^ T0 T1	0 (0–600) 0 (0–2000)
Metamyelocytes/ mm^3^ T0 T1	0 (0–1200) 0 (0–2000)
Bands/ mm^3^ T0 T1	0 (0–4700) 0 (0–8300)
Neutrophils/ mm^3^ T0 T1	5950 (0–22600) 6750 (0–32200)
Lymphocytes/ mm^3^ T0 T1	3000 (0–20500) 2600 (0–23300)
Platelet count/ mm^3^ T0 T1	234000 (11000–873000) 223500 (6000–845000)

Data are expressed as median (range) or n (%). PRISM, Pediatric Risk of Mortality; PELOD, Pediatric Logistic Organ Dysfunction; T0, 72 hours before blood culture collection; T1, day of blood culture collection.

Table 4 shows the results of the analysis of the four models developed in the study. All the models had 100% sensitivity and showed a high accuracy, precision and specificity for the prediction of BSI in our study population. The machine committee model showed the best performance, with the highest accuracy, precision and specificity, compared with the others.

**Table 4:** Accuracy, precision, sensitivity and specificity of the models.

Model	Accuracy	Precision	Sensitivity	Specificity
Machine Committee	99.33	98.89	100	98.46
Logistic Regression	96.64	93.23	100	93.27
Classifier with Logistic Regression performing Feature Selection Pipeline	97.98	95.96	100	96.32
Gradient Boosting Decision Tree	98.64	97.46	100	97.16

Data are expressed as %.

## Discussion

We developed four machine learning models using only routinely collected demographic, clinical and laboratory data, with excellent accuracy and precision, and high sensitivity and specificity for the diagnosis of BSI. Among the developed models, the machine committee model showed the highest accuracy, precision, and specificity, all above 98%. Moreover, it displayed a sensitivity of 100%. Therefore, this model could be used as a decision-support tool to alert the medical team to the risk of BSI in pediatric critically ill patients. As we used simple and widely available data, our model could be also applicable to PICUs from low-resource settings.

Because BSIs are associated with increased morbidity and mortality, early diagnosis and appropriate treatment are paramount to prevent unfavorable outcomes. Thus, a model with high sensitivity and high precision may be a very helpful tool for clinical decision-making. The trained model could be programmed to automatically perform periodic evaluations when new input data is available. With each new set of data, the model could generate an alert in the electronic medical record indicating the probability that the patient has a positive diagnosis for the trained disease. In addition, with each set of new positive and negative measurements that are independently verified and validated by the medical team, the model could receive new data as feedback and, hence, it might be retrained for better performance. After each new retraining, the model gets better at predicting the outcome of interest, thus making improved predictions.

A recent study that used a generalized linear model framework for model generation, which included several clinical, laboratory and treatment data, showed that the model identified 25% of positive blood cultures with a false positive rate of 0.11% in patients following surgery for congenital heart disease [13]. Moreover, a random forest model using urinalysis, white blood cell count, absolute neutrophil count, and procalcitonin showed a sensitivity of 99% and a specificity of 75% for risk stratification of young infants for serious bacterial infection, defined as bacteremia, bacterial meningitis or culture-positive urinary tract infection [25]. In addition, a study that included adult and pediatric patients found an area under the receiver operating characteristic curve of 0.82 for a random forest model for risk prediction of central line-associated BSI. In this study, patient age and device days were the most reliable variable to predict BSI [12]. A study in adults showed that a machine learning model mainly based in the trends of time-series variables, including laboratory results and vital signs, achieved high performance in the prediction of ICU-acquired BSI [10]. In addition, a machine learning technique based on a neural network that used nine clinical parameters measured over time showed a good predictive ability for BSI in critically ill adults [11]. However, neither study in pediatric patients using machine learning for the prediction of BSI included time series data. Conversely, our study included vital signs and laboratory data variation over time, which allowed the model to compare the same patient at two time points and to identify temporal changes that could be suggestive of the diagnosis of BSI.

### Limitations

Our study has some limitations, including its retrospective nature and the fact that it was developed in a single center, which limits its generalizability. In addition, our model has not been implemented at the bedside to verify whether its use will have an impact on patients’ outcomes. Therefore, our model should be prospectively evaluated to check its clinical applicability and potential to improve medical care.

## Conclusions

We developed a machine learning model using routinely collected demographic, clinical and laboratory data, that was able to detect BSI with excellent accuracy and precision, and high sensitivity and specificity. The inclusion of vital signs and laboratory data variation over time allowed the model to identify temporal changes that could be suggestive of the diagnosis of BSI. Our model might help the medical team in clinical-decision making by creating an alert in the electronic medical record, indicating the risk of BSI. This tool may allow early antimicrobial initiation, which can contribute to improved patients’ outcomes.

## Supporting information

S1 Checklist (PDF)

S1 Dataset (PDF)

## References

[1] Timsit JF, Ruppé E, Barbier F, Tabah A, Bassetti M. Bloodstream infections in critically ill patients: an expert statement. Intensive Care Med. 2020;46(2):266–284. doi: 10.1007/s00134-020-05950-6 32047941 PMC7223992

[2] Ista E, van der Hoven B, Kornelisse RF, van der Starre C, Vos MC, Boersma E, et al. Effectiveness of insertion and maintenance bundles to prevent central-line-associated bloodstream infections in critically ill patients of all ages: a systematic review and meta-analysis. Lancet Infect Dis. 2016;16(6):724–734. doi: 10.1016/S1473-3099(15)00409-0 26907734

[3] Haque M, Sartelli M, McKimm J, Abu Bakar M. Health care-associated infections—an overview. Infect Drug Resist. 2018;11:2321–2333. doi: 10.2147/IDR.S177247 30532565 PMC6245375

[4] Bennett EE, VanBuren J, Holubkov R, Bratton SL. Presence of invasive devices and risks of healthcare-associated infections and sepsis. J Pediatr Intensive Care. 2018;7(4):188–195. doi: 10.1055/s-0038-1656535 31073493 PMC6506685

[5] Edwards JD, Herzig CT, Liu H, Pogorzelska-Maziarz M, Zachariah P, Dick AW, et al. Central line-associated blood stream infections in pediatric intensive care units: Longitudinal trends and compliance with bundle strategies. Am J Infect Control. 2015;43(5):489–93. doi: 10.1016/j.ajic.2015.01.006 25952048 PMC4430334

[6] Niedner MF, Huskins WC, Colantuoni E, Muschelli J, Harris JM 2nd, Rice TB, et al. Epidemiology of central line-associated bloodstream infections in the pediatric intensive care unit. Infect Control Hosp Epidemiol. 2011;32(12):1200–8. doi: 10.1086/662621 22080659

[7] Chesshyre E, Goff Z, Bowen A, Carapetis J. The prevention, diagnosis and management of central venous line infections in children. J Infect. 2015;71 Suppl 1:S59–75. doi: 10.1016/j.jinf.2015.04.029 25934326

[8] Savage RD, Fowler RA, Rishu AH, Bagshaw SM, Cook D, Dodek P, et al. The effect of inadequate initial empiric antimicrobial treatment on mortality in critically ill patients with bloodstream infections: a multi-centre retrospective cohort study. PLoS One. 2016;11(5):e0154944. doi: 10.1371/journal.pone.0154944 27152615 PMC4859485

[9] Eliakim-Raz N, Bates DW, Leibovici L. Predicting bacteraemia in validated models—a systematic review. Clin Microbiol Infect. 2015;21(4):295–301. doi: 10.1016/j.cmi.2015.01.023 25677625

[10] Roimi M, Neuberger A, Shrot A, Paul M, Geffen Y, Bar-Lavie Y. Early diagnosis of bloodstream infections in the intensive care unit using machine-learning algorithms. Intensive Care Med. 2020;46(3):454–462. doi: 10.1007/s00134-019-05876-8 31912208

[11] Van Steenkiste T, Ruyssinck J, De Baets L, Decruyenaere J, De Turck F, Ongenae F, et al. Accurate prediction of blood culture outcome in the intensive care unit using long short-term memory neural networks. Artif Intell Med. 2019;97:38–43. doi: 10.1016/j.artmed.2018.10.008 30420241

[12] Beeler C, Dbeibo L, Kelley K, Thatcher L, Webb D, Bah A, et al. Assessing patient risk of central line-associated bacteremia via machine learning. Am J Infect Control. 2018;46(9):986–991. doi: 10.1016/j.ajic.2018.02.021 29661634

[13] Bonello K, Emani S, Sorensen A, Shaw L, Godsay M, Delgado M, et al. Prediction of impending central-line-associated bloodstream infections in hospitalized cardiac patients: development and testing of a machine-learning model. J Hosp Infect. 2022;127:44–50. doi: 10.1016/j.jhin.2022.06.003 35738317

[14] Shah N, Arshad A, Mazer MB, Carroll CL, Shein SL, Remy KE. The use of machine learning and artificial intelligence within pediatric critical care. Pediatr Res. 2023;93(2):405–412. doi: 10.1038/s41390-022-02380-6 36376506 PMC9660024

[15] Kirk D, Kok E, Tufano M, Tekinerdogan B, Feskens EJM, Camps G. Machine Learning in Nutrition Research. Adv Nutr. 2022 Dec 22;13(6):2573–2589. doi: 10.1093/advances/nmac103 36166846 PMC9776646

[16] Pollack MM, Patel KM, Ruttimann UE. PRISM III: an updated Pediatric Risk of Mortality score. Crit Care Med. 1996;24(5):743–52. doi: 10.1097/00003246-199605000-00004 8706448

[17] Leteurtre S, Martinot A, Duhamel A, Proulx F, Grandbastien B, Cotting J, et al. Validation of the paediatric logistic organ dysfunction (PELOD) score: prospective, observational, multicentre study. *Lancet*. 2003;362(9379):192–197. doi: 10.1016/S0140-6736(03)13908-6 12885479

[18] Bose E, Hravnak M, Clermont G. Vector Auto-Regressive (VAR) model for exploring causal dynamics of cardiorespiratory instability. Critical Care Med. 2014;42(12):A1428–A1429 doi: 10.1097/01.ccm.0000457780.29828.f7

[19] Alanazi HO, Abdullah AH, Qureshi KN, Ismail AS. Accurate and dynamic predictive model for better prediction in medicine and healthcare. Ir J Med Sci. 2018;187(2):501–513. doi: 10.1007/s11845-017-1655-3 28756541

[20] Bagley SC, White H, Golomb BA. Logistic regression in the medical literature: standards for use and reporting, with particular attention to one medical domain. J Clin Epidemiol. 2001;54(10):979–85. doi: 10.1016/s0895-4356(01)00372-9 11576808

[21] Pedregosa F, Varoquaux G, Gramfort A, Michel V, Thirion B, Grisel O, et al. Scikit-learn: Machine learning in Python. Journal of Machine Learning Research. 2011;12:2825–2830.

[22] Joksas D, Freitas P, Chai Z et al. Committee machines—a universal method to deal with non-idealities in memristor-based neural networks. Nat Commun. 2020; 11:4273. doi: 10.1038/s41467-020-18098-0 32848139 PMC7450095

[23] https://www.gstatic.com/education/formulas2/553212783/en/logistic_function.svg [Accessed on January 16, 2024].

[24] https://scikit-learn.org/stable/modules/tree.html [Accessed on January 16, 2024].

[25] Ramgopal S, Horvat CM, Yanamala N, Alpern ER. Machine learning to predict serious bacterial infections in young febrile infants. Pediatrics. 2020;146(3):e20194096. doi: 10.1542/peds.2019-4096 32855349 PMC7461239

