# Supplementary material for: A machine learning model for the early diagnosis of bloodstream infection in patients admitted to the pediatric intensive care unit
Source: PLoS One. 2024 May 1;19(5):e0299884. doi: 10.1371/journal.pone.0299884 (PMC11062549; doi:10.1371/journal.pone.0299884)
Supplement: S1 Dataset — (PDF) [file pone.0299884.s002.pdf]

Patient	Age (months)	Weight (kg)	Weight z-score	Height (m)	BMI (kg/m2)	BMI z-score	PRISM	PELOD 48h	Comorbidity	Intravenous device
1	26	12.4	0	0.73	23.1	2	6	0	1	PICC
2	7	5.2	-3	0.47	23.5	3	17	0	1	CVC
3	6	4.4	-3	0.37	32.1	3	10	1	1	PICC
4	1	2.9	-2	0.48	12.7	-1	17	22	1	PICC
5	96	21	-1	1.3	12.4	-2	8	1	1	CVC
6	4	6	0	0.56	19.13	1	10	1	1	CVC
7	1	4	0	0.51	15.3	0	15	12	0	Peripheral vein
8	9	9	0	0.68	19.4	1	22	1	1	CVC
9	2	5	0	0.54	17.1	0	5	1	0	Peripheral vein
10	1	5	0	0.54	17.1	0	5	1	0	Peripheral vein
11	1	2.5	-2	0.46	11.8	-1	13	1	1	CVC
12	19	5.2	-3	0.71	10.3	-3	11	10	1	CVC
13	2	4	-2	0.54	13.7	-1	12	1	1	CVC
14	1	4	0	0.5	16	0	4	10	1	CVC
15	22	7.5	-3	78.5	12.1	-3	9	1	1	Peripheral vein
16	22	7.5	-3	78.5	12.1	-3	9	1	1	PICC
17	104	28	0	missing	missing	missing	11	1	1	PICC
18	20	8	-3	0.725	15.2	0	10	1	1	Totally implantable CVC
19	192	48	missing	1.6	18.75	0	8	0	1	Peripheral vein
20	126	37	0	missing	missing	missing	7	11	1	Peripheral vein
21	1	3.1	-2	0.5	12.4	-2	19	11	1	Peripheral vein
22	4	3.92	-3	0.49	16.3	0	9	1	1	Peripheral vein
23	1	2.5	-3	0.43	13.5	0	12	0	1	Peripheral vein
24	11	9	0	0.74	16.4	0	12	10	1	CVC
25	14	9	0	0.74	16.4	0	0	0	1	Totally implantable CVC
26	2	4	-2	0.55	13.22	-2	7	1	1	CVC
27	16	9.8	0	0.76	16.9	0	5	0	1	Peripheral vein
28	16	5.7	-3	0.7	11.6	-3	16	1	1	PICC
29	0	2.94	0	0.475	13	0	5	0	1	PICC
30	4	5.8	0	0.6	16.1	0	8	1	1	CVC
31	4	5.8	0	0.6	16.1	0	8	1	1	CVC
32	16	9.1	0	0.75	16.1	0	2	0	1	CVC
33	0	3.9	0	0.5	15.6	0	12	2	1	Umbilical catheter
34	7	6.1	-3	0.56	19.45	1	8	1	1	PICC
35	1	3.2	-2	0.49	13.3	-1	14	11	0	Peripheral vein
36	0	2.9	0	0.48	12.5	0	14	12	1	PICC
37	0	3.5	0	0.49	14.5	0	6	11	1	PICC
38	3	2.25	-3	0.565	7.1	-3	16	11	1	CVC

39	5	2.5	-3	0.565	7.9	-3	16	10	1	PICC
40	36	20.4	2	1.02	19.6	2	5	1	1	CVC
41	5	6	0	0.65	14.2	-1	14	11	1	CVC
42	2	3.9	-2	0.5	15.6	0	21	12	1	CVC
43	192	39	missing	1.63	14.69	-3	0	0	1	Totally implantable CVC
44	2	3.8	-2	0.5	15.2	0	5	1	1	PICC
45	2	4.1	-2	0.53	14.5	-1	8	1	1	CVC
46	12	5.2	-3	0.64	12.6	-3	7	1	1	Peripheral vein
47	60	22	0	1.12	17.5	1	33	3	1	PICC
48	2	5	0	0.5	20	2	8	11	1	PICC
49	84	28	1	1.2	19.4	2	6	1	1	CVC
50	2	4.6	0	0.53	16.3	0	3	1	0	CVC
51	192	50	missing	1.67	17.9	-1	17	3	1	CVC
52	18	11.2	0	0.815	16.8	0	18	11	1	CVC
53	11	9.4	0	0.67	20.9	2	16	0	1	PICC
54	4	4.3	-3	0.52	15.2	-1	24	1	1	CVC
55	1	3.2	-2	0.48	13.8	-1	11	11	1	PICC
56	4	4.5	-3	0.48	19.5	1	11	11	1	Peripheral vein
57	0	3.8	0	0.46	17.9	3	13	11	1	CVC
58	3	4.5	-2	0.55	14.8	-1	3	1	1	CVC
59	0	3.1	0	0.46	14.6	1	14	11	1	CVC
60	27	10	0	0.73	18.7	2	24	2	1	Peripheral vein
61	180	53	missing	1.6	20.7	0	0	0	1	CVC
62	7	7.6	0	0.63	19.1	1	18	11	1	CVC
63	7	7.6	0	0.63	19.1	1	18	11	1	CVC
64	18	9.6	0	0.75	17.4	0	9	1	1	CVC
65	5	7.2	0	0.65	17	0	11	1	1	CVC
66	4	5.1	0	missing	missing	missing	7	1	1	Peripheral vein
67	32	10.3	-2	missing	missing	missing	9	11	1	CVC
68	2	3.6	-3	0.5	14.4	-1	13	0	1	CVC
69	2	3.6	-3	0.5	14.4	-1	18	11	0	PICC
70	65	14	-2	1.1	11.5	-3	16	1	0	CVC
71	2	2.8	-3	0.47	12.6	-3	26	1	1	Peripheral vein
72	0	2.5	0	0.44	12.9	0	26	11	1	CVC
73	4	4.6	-3	0.62	11.9	-3	1	1	1	Peripheral vein
74	156	94	missing	1.65	34.5	3	0	0	1	Totally implantable CVC
75	16	9	0	0.76	15.5	0	9	1	0	Peripheral vein
76	0	2.8	0	0.5	11.2	-1	6	11	1	PICC

Femoral site	ΔT IV-line insertion to blood culture collection (days)	Duration of total parenteral nutrition (days)	C-reactive protein 72 h before blood culture collection (mg/dl)
0	14	790	0.07
0	6	0	3.71
0	5	0	0.09
0	15	0	4.08
0	4	4	0
0	7	0	1.68
0	0	0	2.58
1	4	0	1.1
0	0	0	1.1
0	0	0	3.19
0	9	0	1.94
0	10	7	12
0	16	0	22.18
0	15	0	3.73
0	0	0	0
0	40	13	0
1	39	0	8.04
0	0	0	0
0	0	0	33.38
0	0	0	26.48
0	0	0	19.84
0	0	0	1.45
0	0	0	0.86
1	10	0	5.97
0	5	0	0
1	22	0	0.4
0	0	0	34.73
0	2	0	4.01
0	1	0	0.01
1	7	0	0.15
1	8	0	0.63
0	18	1	28.8
0	7	0	1.48
1	9	21	1.66
0	0	0	20.85
0	7	0	1.5
1	3	0	1
0	4	0	21.88

1	29	0	2
1	4	0	27.67
1	2	0	0.17
0	11	0	5.17
0	180	0	0
1	1	14	5.92
0	2	0	0
0	0	0	2.3
0	13	0	3.65
1	14	0	10.7
1	10	0	0.14
0	10	0	0.79
0	1	0	6.77
1	11	0	1.76
0	3	3	42.79
0	3	0	15.4
0	4	0	3.11
0	0	0	3.69
0	1	0	11.83
0	5	0	0.01
0	4	0	0.67
0	0	0	6.48
0	10	0	3.18
0	9	0	16.55
0	10	0	7.78
0	14	13	7.47
1	10	13	16.02
0	0	0	7.49
0	14	0	1.79
0	6	0	6.93
0	8	0	1.91
1	22	0	4.45
0	0	0	35.8
0	11	0	2.1
0	0	0	0.47
0	17	0	4.83
0	0	0	23.1
0	4	4	0

White blood cell count 72 h before blood culture collection (/mm3)	Myelocyte count 72h before blood culture collection (/mm3)	Metamyelocyte count 72 h before blood culture collection (/mm3)
9200	0	0
12700		
8200	0	0
10700	0	0
0	0	0
8900	0	0
1900	0	0
10000	0	0
31800	0	0
30700	0	0
6900	0	0
11000	0	0
16000	0	0
13400	0	0
9200	0	0
0	0	0
7400	0	0
16700	0	0
12900	0	0
19800	0	0
1200	0	0
8200	0	0
10600	0	0
700	0	0
4800	0	0
11500	0	0
15900	0	0
8900	0	0
19700	0	0
5200	0	0
5900	0	0
22600	0	0
8400	0	0
10800	0	0
9000	0	0
11600	0	0
13000	0	0
6400	600	300

13300	0	0
9600	0	0
7500	0	0
4700	0	0
13700	0	0
13700	0	0
2500	0	0
9200	0	0
16300	0	0
11600	0	0
12100	0	0
14300	0	0
6200	0	0
8900	0	0
3500	0	0
14600	600	1200
15100	0	0
11300	0	0
8700	0	0
10200	0	0
13300	0	0
10500	0	0
12200	0	0
8400	0	0
12600	0	0
22200	0	0
16100	0	0
15300	0	0
300	0	0
7700	0	0
10700	0	0
8200	0	0
37400	0	0
25800	0	0
10300	0	0
11900	0	0
6600	0	0
14700	0	0

Bands count 72 h before blood culture collection (/mm3)	Neutrophil count 72 h before blood culture collection (/mm3)	Lymphocyte count 72 h before blood culture collection (/mm3)
0	3700	4400
2200	5000	5500
0	3200	4000
200	7000	2200
0	0	0
0	2800	5500
0	0	0
0	4000	5200
2000	22600	5700
600	10700	17500
100	1400	4800
0	8100	2000
0	8900	5600
0	8600	3000
0	8800	100
0	0	0
0	4200	2200
4700	3500	6000
0	9400	2400
400	15600	3000
0	0	0
0	3400	3700
0	5100	4200
0	100	600
0	2100	2400
0	5300	5300
0	11800	3000
0	6700	2000
0	13800	3900
0	3500	1500
0	500	4800
0	15200	5200
0	3500	3600
0	6700	3200
0	6200	2000
0	6300	4100
0	8100	2500
900	3300	1200

0	9000	3100
0	4100	4300
0	4900	2200
0	2800	1500
0	10300	2200
0	9800	2500
0	200	2200
0	4900	3600
0	12600	2300
0	5900	4600
0	8600	2900
0	6400	6900
700	3900	1000
0	4700	3300
0	1200	2200
700	12000	100
0	10900	2600
0	8100	2000
0	1900	6200
0	3100	6400
0	7200	4000
0	6800	200
0	8300	2600
0	2600	4800
0	6000	5600
1100	16000	3600
0	13800	1500
0	10200	4200
0	100	200
0	5100	1800
0	6200	3300
0	5900	1800
400	14900	20500
1800	18200	3900
0	4300	5200
0	10100	1000
600	1300	4600
0	10600	2500

Platelet count 72 h before blood culture collection (/mm3)	C-reactive protein on blood culture collection day (mg/dl)	White blood cell count on blood culture collection day (/mm3)
184000	0.01	12900
267000	2.47	8900
236000	9.76	10100
274000	25.44	3700
24000	25.99	100
330000	5.87	10200
167000	25.79	3200
56000	7.53	10900
669000	12.62	21300
494000	1.43	44300
232000	1.46	16700
61000	22.55	9100
232000	22.29	32800
300000	6.14	24100
29000	0	100
41000	42.7	100
549000	9.23	7600
429000	27.1	15500
174000	6.92	7200
258000	11.19	26900
407000	33.08	800
220000	8.93	17100
495000	1.21	9800
34000	14.96	800
68000	0.71	2700
457000	4.25	13400
157000	25.18	14100
226000	0.81	6200
337000	0.01	14900
283000	8.8	4800
237000	2.12	9200
812000	24.4	22700
210000	1.51	6300
200000	13.18	12100
11000	23.16	14000
113000	5.04	14900
292000	0.55	12100
74000	13.69	17800

473000	11.99	7300
311000	23.17	17900
218000	4.91	10100
62000	6.27	10600
555000	9.95	11600
304000	6.47	10800
108000	0	1000
536000	18.55	4900
285000	3.35	16500
325000	27.26	19700
375000	6.25	15100
589000	16.06	8900
28000	3.84	6000
178000	1.66	11900
212000	45.22	5800
63000	20.9	42900
397000	24.98	37700
490000	7.32	23700
203000	1.97	21000
524000	10.39	7100
165000	16.92	12000
110000	12.43	5900
622000	6.28	13500
117000	5.96	15800
210000	3.24	20300
630000	20.38	26600
180000	28.04	16900
873000	10.57	29200
64000	15.24	100
174000	30.72	4800
489000	0.55	8900
281000	2.8	6200
342000	10.8	18500
269000	4.5	14700
187000	0.41	9000
108000	1.03	10200
215000	29.4	17500
244000	2.9	8500

Myelocyte count on blood culture collection day (/mm3)	Metamyelocyte count on blood culture collection day (/mm3)	Bands count on blood culture collection day (/mm3)
0	0	0
0	0	0
0	0	0
0	0	0
0	0	0
0	0	0
0	0	120
0	0	0
0	0	1300
0	0	900
0	0	0
0	0	0
0	0	600
0	200	200
0	0	0
0	0	0
0	0	0
0	0	2900
0	0	0
800	800	8300
0	0	0
0	0	0
0	0	0
0	0	0
0	0	0
0	0	0
0	0	0
0	0	0
0	0	0
0	0	0
0	0	0
0	0	2000
0	0	0
0	0	400
0	400	1100
0	0	0
0	0	0
600	1200	2900

0	0	0
0	0	0
0	0	0
0	0	0
0	0	0
0	0	0
0	0	0
0	0	0
0	0	0
0	0	600
0	0	0
0	0	0
100	100	700
0	0	0
0	0	0
2000	2000	2000
0	0	3000
0	800	5000
0	400	700
0	0	0
100	400	600
0	0	0
0	0	0
0	0	0
0	400	800
300	300	1800
1100	1200	1700
0	0	2100
0	0	0
0	0	600
0	0	0
0	0	0
0	0	1000
0	0	0
0	0	0
0	0	0
0	0	1200
0	0	400

Neutrophil count on blood culture collection day (/mm3)	Lymphocyte count on blood culture collection day (/mm3)	Platelet count on blood culture collection day (/mm3)
5100	6300	182000
4200	3900	15000
6500	2200	208000
2600	1000	116000
0	100	22000
6900	2800	394000
600	2200	64000
2000	7200	151000
14600	3700	525000
17500	23300	483000
10100	4600	256000
6600	1100	53000
10900	20600	73000
20700	1400	380000
100	0	9000
100	0	20000
5600	1100	496000
7400	3900	304000
5000	1600	254000
14300	2400	342000
400	400	114000
13500	2500	245000
5100	3800	407000
100	700	51000
1000	1500	73000
7500	4400	610000
9000	3900	381000
2800	2300	250000
9400	3700	372000
2900	1500	181000
400	7500	452000
13600	4100	845000
1800	3700	290000
5900	5200	6000
8900	2500	30000
9700	4100	106000
7100	3400	339000
11400	1400	38000

3800	2400	392000
13600	2900	365000
8000	1700	200000
5300	4400	100000
8900	1700	545000
9100	1400	287000
0	900	33000
2800	1600	403000
12500	2200	329000
11600	5500	261000
11500	2900	233000
3800	4500	344000
4300	500	41000
3900	6400	222000
1600	3100	179000
32200	1700	28000
29800	800	585000
13500	3100	572000
10200	7500	169000
3900	2700	96000
8700	1300	37000
4400	1000	40000
8600	3100	765000
7000	6800	225000
7900	10200	171000
19400	3600	637000
0	0	41000
19500	6500	801000
0	0	22000
1800	2000	298000
3800	3900	454000
5000	900	208000
13800	3500	83000
7400	2500	241000
4700	3600	408000
8900	900	117000
8700	6100	106000
5400	2000	151000
